# The Association of Age-Related and Off-Target Retention with Longitudinal Quantification of [^18^F]MK6240 Tau PET in Target Regions

**DOI:** 10.2967/jnumed.122.264434

**Published:** 2023-03

**Authors:** Cécile Tissot, Stijn Servaes, Firoza Z. Lussier, João Pedro Ferrari-Souza, Joseph Therriault, Pâmela C.L. Ferreira, Gleb Bezgin, Bruna Bellaver, Douglas Teixeira Leffa, Sulantha S. Mathotaarachchi, Mira Chamoun, Jenna Stevenson, Nesrine Rahmouni, Min Su Kang, Vanessa Pallen, Nina Margherita-Poltronetti, Yi-Ting Wang, Jaime Fernandez-Arias, Andrea L. Benedet, Eduardo R. Zimmer, Jean-Paul Soucy, Dana L. Tudorascu, Annie D. Cohen, Madeleine Sharp, Serge Gauthier, Gassan Massarweh, Brian Lopresti, William E. Klunk, Suzanne L. Baker, Victor L. Villemagne, Pedro Rosa-Neto, Tharick A. Pascoal

**Affiliations:** 1 McGill University, Montreal, Quebec, Canada;; 2 McGill University Research Center for Studies in Aging, Montreal, Quebec, Canada;; 3 Departments of Psychiatry and Neurology, University of Pittsburgh School of Medicine, Pittsburgh, Pennsylvania;; 4 Graduate Program in Biological Sciences: Biochemistry, Porto Alegre, Brazil;; 5 Artificial Intelligence and Computational Neurosciences Lab, Sunnybrook Research Institute, University of Toronto, Toronto, Ontario, Canada;; 6 L.C. Campbell Cognitive Neurology Unit, Hurvitz Brain Sciences Program, Sunnybrook Research Institute, University of Toronto, Toronto, Ontario, Canada;; 7 University of Gothenburg, Gothenburg, Sweden;; 8 Department of Pharmacology, Universidade Federal do Rio Grande do Sul, Porto Alegre, Brazil;; 9 Montreal Neurological Institute, Montreal, Quebec, Canada;; 10 Douglas Mental Health Institute, Montreal, Quebec, Canada;; 11 Department of Radiochemistry, Montreal Neurological Institute, Montreal, Quebec, Canada;; 12 Department of Radiology, University of Pittsburgh School of Medicine, Pittsburgh, Pennsylvania; and; 13 Lawrence Berkeley National Laboratory, Berkeley, California

**Keywords:** tau, PET, reference region, off-target binding, [^18^F]MK6240

## Abstract

6-(fluoro-^18^F)-3-(1H-pyrrolo[2,3-c]pyridin-1-yl)isoquinolin-5-amine ([^18^F]MK6240) tau PET tracer quantifies the brain tau neurofibrillary tangle load in Alzheimer disease. The aims of our study were to test the stability of common reference region estimates in the cerebellum over time and across diagnoses and evaluate the effects of age-related and off-target retention on the longitudinal quantification of [^18^F]MK6240 in target regions. **Methods:** We assessed reference, target, age-related, and off-target regions in 125 individuals across the aging and Alzheimer disease spectrum with longitudinal [^18^F]MK6240 SUVs and SUV ratios (SUVRs) (mean ± SD, 2.25 ± 0.40 y of follow-up). We obtained SUVR from meninges, exhibiting frequent off-target retention with [^18^F]MK6240. Additionally, we compared tracer uptake between 37 cognitively unimpaired young (CUY) (mean age, 23.41 ± 3.33 y) and 27 cognitively unimpaired older (CU) adults (amyloid-β–negative and tau-negative, 58.50 ± 9.01 y) to identify possible nonvisually apparent, age-related signal. Two-tailed *t* testing and Pearson correlation testing were used to determine the difference between groups and associations between changes in region uptake, respectively. **Results:** Inferior cerebellar gray matter SUV did not differ on the basis of diagnosis and amyloid-β status, cross-sectionally and over time. [^18^F]MK6240 uptake significantly differed between CUY and CU adults in the putamen or pallidum (affecting ∼75% of the region) and in the Braak II region (affecting ∼35%). Changes in meningeal and putamen or pallidum SUVRs did not significantly differ from zero, nor did they vary across diagnostic groups. We did not observe significant correlations between longitudinal changes in age-related or meningeal off-target retention and changes in target regions, whereas changes in all target regions were strongly correlated. **Conclusion:** Inferior cerebellar gray matter was similar across diagnostic groups cross-sectionally and stable over time and thus was deemed a suitable reference region for quantification. Despite not being visually perceptible, [^18^F]MK6240 has age-related retention in subcortical regions, at a much lower magnitude but topographically colocalized with significant off-target signal of the first-generation tau tracers. The lack of correlation between changes in age-related or meningeal and target retention suggests little influence of possible off-target signals on longitudinal tracer quantification. Nevertheless, the age-related retention in the Braak II region needs to be further investigated. Future postmortem studies should elucidate the source of the newly reported age-related [^18^F]MK6240 signal, and in vivo studies should further explore its impact on tracer quantification.

The accumulation of amyloid-β (Aβ) plaques and hyperphosphorylated tau, forming neurofibrillary tangles (NFTs), is a hallmark of Alzheimer disease (AD) (1) and can be observed in aging and AD dementia (2). The tau levels in the brain are assessed through cerebrospinal fluid and PET imaging. Radiotracers used in PET imaging are considered optimal when they present desirable characteristics such as rapidly equilibrating in vivo kinetics, low off-target retention, no significant lipophilic radiolabeled metabolites able to enter the brain, and high affinity for their target (3).

6-(fluoro-^18^F)-3-(1H-pyrrolo[2,3-c]pyridin-1-yl)isoquinolin-5-amine ([^18^F]MK6240) is a promising tracer allowing for the quantification of fibrillary tau pathology in vivo, with postmortem studies confirming its binding to paired helical fragments of phosphorylated tau (4–7). The tracer binds with high affinity to NFTs, thus making it specific to AD-related tauopathy. As shown in postmortem data, the tracer does not seem to bind to tau aggregates in non-AD tauopathies (5*,*^8^), except in rare frontotemporal dementia mutations associated with brain deposition of NFTs (9). [^18^F]MK6240 allows for the differentiation between cognitively unimpaired (CU), mild cognitive impairment (MCI), and AD subjects (4). Furthermore, [^18^F]MK6240 has been shown to recapitulate in vivo the tau pathologic stages, proposed via postmortem studies by Braak et al. (1*,*^10^*,*^11^).

Despite several favorable features of [^18^F]MK6240, some common challenges in PET studies remain unaddressed for this tracer, such as the choice of a reference region for longitudinal studies and the impact of off-target retention on tracer quantification in target regions (i.e., regions expected to show specific, tau-related retention of [^18^F]MK6240). Postmortem and in vivo studies have indicated that [^18^F]MK6240 has off-target retention in neuromelanin-containing cells (5). Those are regions, such as the substantia nigra, also observed using first-generation tau PET tracers (12). However, [^18^F]MK6240 shows significant off-target retention in the meninges (4*,*^13^), a characteristic that is currently the main concern for accurate quantification of NFTs using this tracer.

As longitudinal tracer quantification is critical for clinical trials using tau PET imaging agents as a possible surrogate marker of tau accumulation, exploring the optimal reference region and the effects of off-target retention on longitudinal [^18^F]MK6240 quantification are crucial (14*,*^15^). Here, we studied longitudinal changes in reference, target, age-related, and off-target regions across diagnostic groups and Aβ status to elucidate the caveats associated with the longitudinal quantification of [^18^F]MK6240.

## MATERIALS AND METHODS

### Participants

We included individuals from the TRIAD cohort (16), with data obtained from December 2017 to November 2021. The study was approved by the Douglas Mental Institute Research Board, and all participants gave written consent. Detailed information gathered from the participants can be found online (https://triad.tnl-mcgill.com/). All participants underwent a complete neuropsychologic evaluation, MRI, and acquisition of both [^18^F]flutafuranol ([^18^F]AZD4694) (Aβ) and [^18^F]MK6240 (tau) PET scans. We used 2 distinct subject samples. To assess age-related off-target retention of [^18^F]MK6240, we included 37 cognitively unimpaired young (CUY) adults (<35 y old) and 27 cognitively unimpaired older (CU) adults (40–65 y old), both presenting no AD-related pathology (Aβ and tau); this sample was called the age-related sample and included only cross-sectional data. The cutoff for a positive Aβ status was a [^18^F]AZD4694 global PET SUVR of less than 1.55 (17), whereas the cutoff for a positive tau status was a [^18^F]MK6240 temporal meta–region of interest with an SUVR of less than 1.24, as previously described (18). The longitudinal sample comprised 125 individuals (11 CUY, 66 CU Aβ-negative [Aβ−], 17 CU Aβ-positive [Aβ+], and 31 cognitively impaired [CI] Aβ+, including 22 multidomain amnestic MCI and 9 AD) who underwent a follow-up assessment between 1.5 and 3.5 y after their baseline assessment. Baseline diagnosis was used in the analyses, after clinical assessments, based on the Mini-Mental State Examination (MMSE) and Clinical Dementia Rating (CDR) scoring, according to the criteria of the National Institute of Aging Alzheimer’s Association (19). CU individuals did not have objective impairment, had an MMSE score of 26 or more, and had a CDR score of 0 (20). Individuals diagnosed with MCI had subjective or objective cognitive impairment and had relatively preserved activities of daily life, defined as an MMSE score of 26 or above and a CDR of 0.5 (21). Dementia due to AD was defined as an MMSE score of less than 26 and a CDR score of 0.5 or more. No participant met the criteria for another neurologic or major neuropsychiatric disorder after a clinical interview performed by a trained physician.

### PET Image Processing

Participants underwent T1-weighted MRI (3-T; Siemens), as well as [^18^F]MK6240 tau PET and [^18^F]AZD4694 Aβ PET using the same brain-dedicated Siemens High Resolution Research Tomograph. [^18^F]MK6240 images were acquired 90–110 min after tracer injection and reconstructed using an ordered-subset expectation maximization algorithm on a 4-dimensional volume with 4 frames (4 × 300 s) (4). [^18^F]AZD4694 images were acquired 40–70 min after tracer injection and reconstructed with the same ordered-subset expectation maximization algorithm with 3 frames (3 × 600 s) (4). Each PET acquisition concluded with a 6-min transmission scan with a rotation ^137^Cs point source for attenuation correction. Images were further corrected for motion, decay, dead time, and random and scattered coincidences. SUV images were calculated considering the injected radionuclide dose and weight of each participant $\left(\frac{\text{PET}}{\text{dose/weight}}\right)$. Injected dose and weight information can be found in Supplemental Table 1 (supplemental materials are available at http://jnm.snmjournals.org). SUVs were extracted from the inferior cerebellar gray matter (CG), superior CG, crus I, and full CG. Information on the masks can be found in Supplemental Table 2. SUV ratio (SUVR) images were generated using the inferior CG as the reference region for [^18^F]MK6240 and the full CG for [^18^F]AZD4694. Finally, images were spatially smoothed to achieve a final gaussian kernel of 8 mm in full width at half maximum (22*,*^23^). Supplemental Table 3 outlines the mean sensitivity of the scanner at baseline and at follow-up visits. The meninges were not masked at any step of the processing. A population-based meningeal mask was created with the Montreal Neurological Institute MINC tool kit as the region having more than a 90% probability of being part of either telencephalon or cerebellar meninges in CUY individuals (Supplemental Fig. 1). In addition, we categorized individuals as having high or low meningeal retention, based on the meningeal SUV median of the population. SUVRs in Braak regions were extracted following the method of Pascoal et al. (10). Additionally, the Desikan–Killiany–Tourville atlas (24) was used to obtain [^18^F]MK6240 SUVR from the putamen and the pallidum. A global [^18^F]AZD4694 SUVR was estimated by averaging the SUVR from the precuneus, prefrontal, orbitofrontal, parietal, temporal, anterior, and posterior cingulate cortices (25). The cutoff to classify participants as Aβ+ or Aβ− was a global SUVR of 1.55 (17). The Montreal Neurological Institute MINC tool kit was used to calculate average images of [^18^F]MK6240 retention.

### Statistical Analysis

R statistical software (version 4.0.0) was used to perform nonimaging statistical analyses. *t* testing or ANOVA was used for continuous variables, and χ^2^ or Fisher testing was used for categoric variables for demographic information when appropriate. The coefficient of variation was calculated as the group SD divided by the mean. Longitudinal change (Δ) was calculated as follows: $\frac{\left(\text{follow-up}\;\text{SUV}(\text{R})\;-\;\text{baseline}\;\text{SUV}(\text{R})\right)}{\text{time}\;(\text{y})}.$ Associations between changes in biomarkers were assessed with Pearson correlation. Voxelwise statistical comparisons were conducted using VoxelStats, a statistical tool box implemented in MATLAB (MathWorks) (26). Age-related retention was evaluated at the voxel level using a 2-sided *t* test between CUY and CU Aβ− and tau-negative elderly individuals aged from 40 to 65 y (Table 1). False-discovery-rate correction was applied with a voxel-level correction of *P* < 0.05.

**TABLE 1.** Demographics: Dataset Used to Assess Age-Related Retention

Parameter	CUY (*n* = 37)	CU Aβ− < 65 y (*n* = 27)	*P*
Age (y)	23.41 (3.3)	58.09 (9.2)	<0.001
Female	24 (64.9%)	13 (48.1%)	0.28
Education (y)	16.91 (2.5)	15.41 (3.4)	0.0167
MMSE	29.86 (0.4)	29.22 (0.9)	<0.001
CDR	0.00 (0.0)	0.00 (0.0)	Not applicable

Qualitative data are number and percentage; continuous data are mean and SD.

## RESULTS

### Participants

In the age-related group analyses, comparing CUY and CU older adults who were both Aβ− and tau-negative, we observed no significant difference in sex and years of education. By definition, subjects had a significant difference in age. We also observed a small but significant difference in the MMSE score, with the CU older adults having a slightly lower score (Table 1). In the longitudinal dataset, as expected, we observed significant differences in age, MMSE scores, and CDR scores across groups. There was no difference in years of education; however, a small but significant difference was observed regarding sex, with more women in the CUY and CU Aβ+ groups (Table 2).

**TABLE 2.** Longitudinal Dataset

Parameter	CUY	CU Aβ−	CU Aβ+	CI Aβ+	*P*
Age (y)	22.65 (1.9)	68.44 (9.8)	74.91 (5.1)	71.44 (5.3)	<0.001
Female (%)	8 (72.7%)	41 (62.1%)	14 (82.4%)	14 (45.2%)	0.0668
Education (y)	16.18 (1.7)	16.27 (4.1)	14.65 (2.4)	15.13 (3.4)	0.2
MMSE	29.82 (0.6)	29.21 (1.1)	29.00 (1.0)	26.06 (4.6)	<0.001
CDR	0.00 (0.0)	0.00 (0.0)	0.00 (0.0)	0.65 (0.4)	<0.001

Qualitative data are number and percentage; continuous data are mean and SD.

### Assessment of Stability of Reference Regions over Time for Use in Longitudinal Studies

**FIGURE 1.**
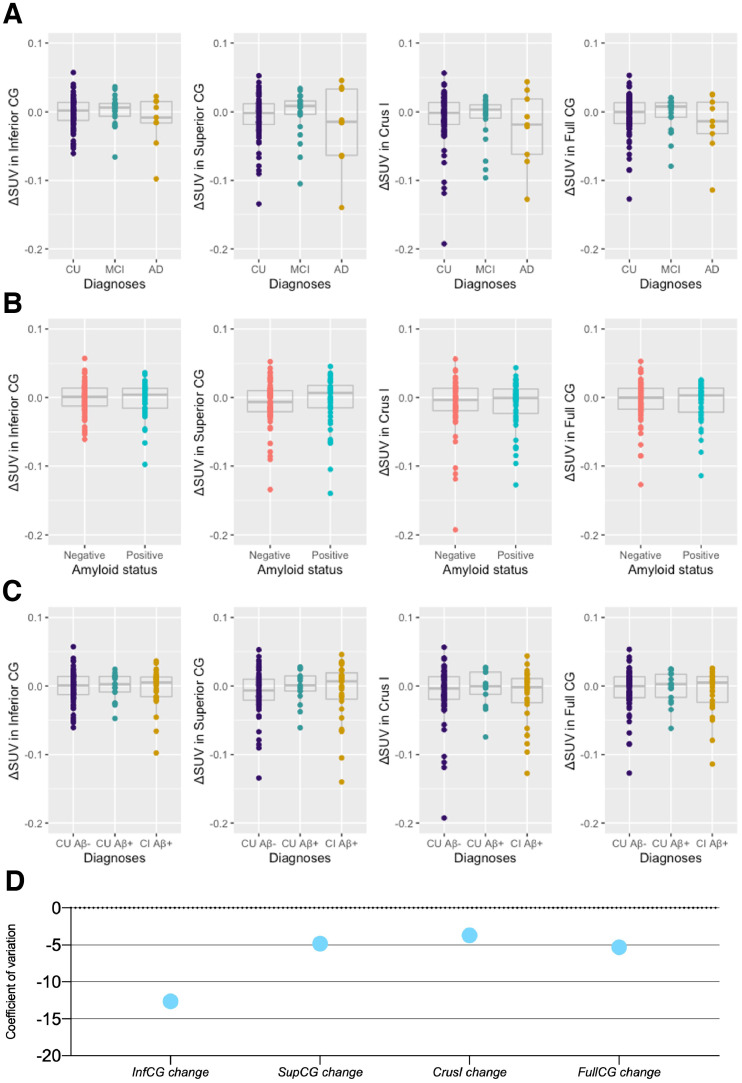
Annualized longitudinal changes in [^18^F]MK6240 SUV in cerebellar candidate reference regions. (A–C) ΔSUV of [^18^F]MK6240 did not significantly differ across diagnosis (A), Aβ status (B), and diagnosis and Aβ status (C). (D) Coefficient of variation of longitudinal changes in SUV within reference regions. Δ = change calculated as $\frac{\left(\text{follow-up}\;\text{SUV}\;-\;\text{baseline}\;\text{SUV}\right)}{\text{time}\;(\text{y})}.$ Inf = inferior; sup = superior.

Our first objective was to ascertain the reference region appropriateness for longitudinal quantification of [^18^F]MK6240. Using ΔSUV over time, we tested the stability of SUV over the time frame of our study in the inferior CG, superior CG, cerebellar crus I, and full CG. No significant differences in longitudinal changes were observed when individuals were separated on the basis of their clinical diagnosis (Fig. 1A), Aβ status (Fig. 1B), or both (Fig. 1C). The mean and SD for ΔSUV can be found in Supplemental Table 4. Coefficients of variation for [^18^F]MK6240 ΔSUV were similar, being highest for inferior CG longitudinal change (−12.64) and lowest for crus I (−3.71) (Fig. 1D; Table 3). We observed no significant variability in SUV (i.e., between baseline and follow-up measures) in the assessed reference regions (Table 4; Supplemental Table 5).

**TABLE 3.** Coefficient of Variation of Cerebellar Regions Across Diagnosis, at Baseline, and at Follow-Up and Longitudinal Changes

Region	All	CU Aβ−	CU Aβ+	CI Aβ+
Inferior CG BL	0.19	0.18	0.14	0.22
Superior CG BL	0.27	0.24	0.19	0.32
Crus I BL	0.25	0.26	0.19	0.28
Full CG BL	0.22	0.22	0.19	0.24
Inferior CG FU	0.17	0.18	0.16	0.17
Superior CG FU	0.25	0.21	0.21	0.29
Crus I FU	0.22	0.21	0.22	0.25
Full CG FU	0.20	0.20	0.21	0.19
ΔInferior CG	−12.64	−14.35	−11.12	−11.03
ΔSuperior CG	−4.83	−3.79	−52.03	−5.05
ΔCrus I	−3.71	−3.84	−7.48	−2.93
ΔFull CG	−5.30	−5.47	−9.63	−4.28

BL = baseline; FU = follow-up.

Longitudinal change was calculated using formula: Δ = $\frac{\left(\text{follow-up}\;\text{SUV}\;-\;\text{baseline}\;\text{SUV}\right)}{\text{time}\;(\text{y})}.$

**TABLE 4.** *P* Values of 2-Tailed *t* Test Between Baseline and Follow-up SUV Across Cerebellar Regions in Individuals Categorized by Diagnosis

Region	All	CU Aβ−	CU Aβ+	CI Aβ+
Inferior CG	0.67	0.81	0.72	0.83
Superior CG	0.27	0.21	0.94	0.66
Crus I	0.09	0.18	0.72	0.31
Full CG	0.25	0.40	0.76	0.46

The supplemental material displays the cross-sectional differences in those SUVs. In the cross-sectional analysis, only superior CG (CU–AD, *P* < 0.001; MCI–AD, *P* < 0.001) and crus I (CU–AD, *P* = 0.046; MCI–AD, *P* = 0.051) presented significant differences between diagnostic groups after correction for multiple comparisons (Supplemental Fig. 2). Cross-sectional coefficients of variation for reference regions are reported in Supplemental Figure 3.

### Meningeal and Age-Related Retentions

**FIGURE 2.**
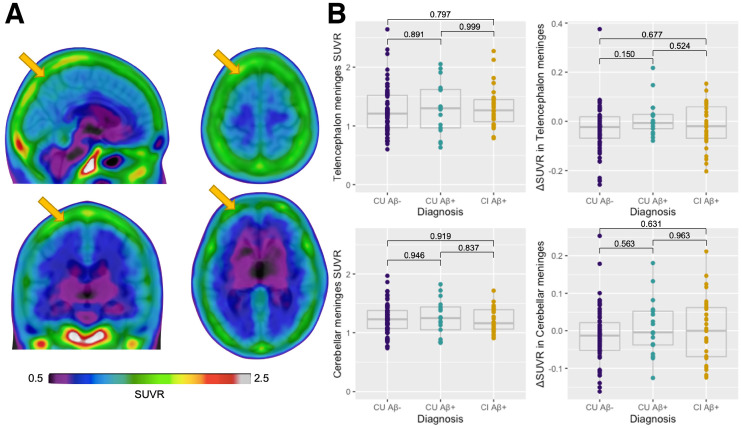
Cross-sectional and longitudinal meningeal [^18^F]MK6240 SUVR across groups. (A) Representative [^18^F]MK6240 average SUVR image in CUY individuals. (B) Cross-sectional and longitudinal changes in [^18^F]MK6240 (ΔSUVR) in telencephalon and cerebellar meninges showing no significant differences depending on diagnosis and Aβ status. Δ = change calculated as $\frac{\left(\text{follow-up}\;\text{SUVR}\;-\;\text{baseline}\;\text{SUVR}\right)}{\text{time}\;(\text{y})}.$ Yellow arrows indicate meninges.

Figure 2A represents the average [^18^F]MK6240 SUVRs in the CUY group. First, we detected a strong correlation between telencephalon and cerebellar meninges, cross-sectionally and longitudinally (Supplemental Fig. 4). We observed no significant difference between diagnostic groups in the telencephalon meninges cross-sectionally (CU Aβ− vs. CU Aβ+, *P* = 0.891; CU Aβ− vs. CI Aβ+, *P* = 0.797; CU Aβ+ vs. CI Aβ+, *P* = 0.999) or longitudinally (CU Aβ− vs. CU Aβ+, *P* = 0.150; CU Aβ− vs. CI Aβ+, *P* = 0.677; CU Aβ+ vs. CI Aβ+, *P* = 0.524). Similarly, no differences were observed in the cerebellar meninges either cross-sectionally (CU Aβ− vs. CU Aβ+, *P* = 0.946; CU Aβ− vs. CI Aβ+, *P* = 0.919; CU Aβ+ vs. CI Aβ+, *P* = 0.837) or longitudinally (CU Aβ− vs. CU Aβ+, *P* = 0.563; CU Aβ− vs. CI Aβ+, *P* = 0.631; CU Aβ+ vs. CI Aβ+, *P* = 0.963) (Fig. 2B). Finally, SUVRs in the meninges were higher in women than men transversally but not longitudinally (Supplemental Fig. 5).

**FIGURE 3.**
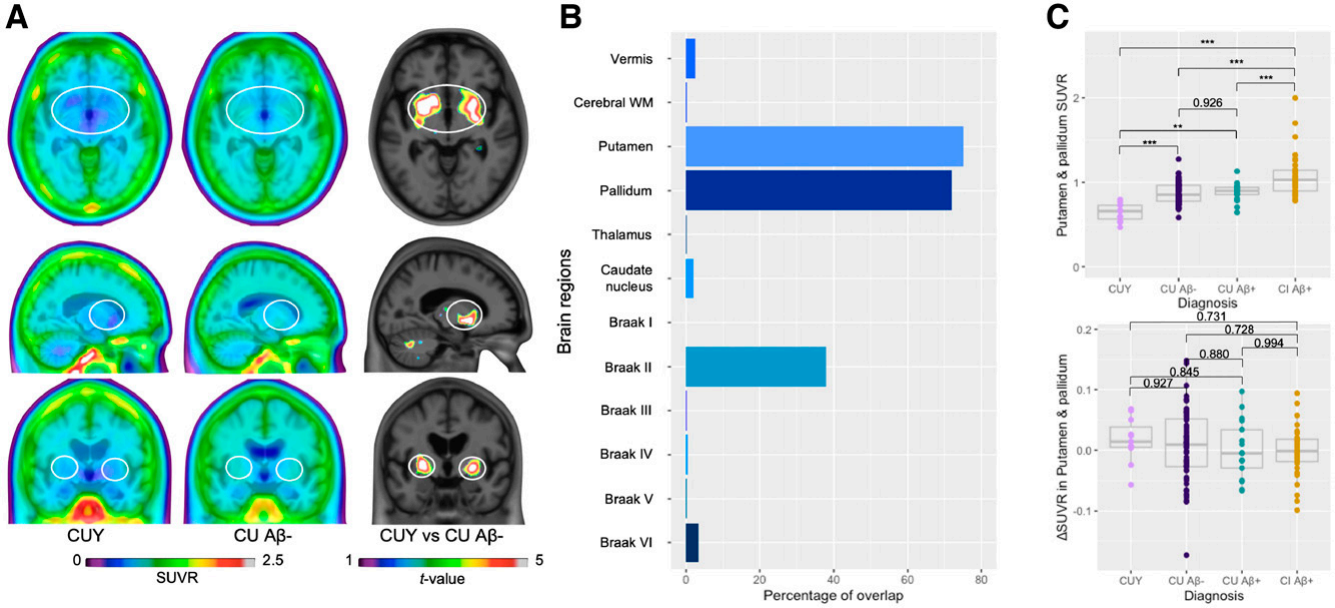
[^18^F]MK6240 age-related retention. (A) [^18^F]MK6240 average SUVR images in CUY and CU Aβ− individuals did not seem to show strong differences visually. *t* test between 2 groups depicts age-related retention in putamen, pallidum, cortical regions, and cerebellar white matter. (B) Percentage of overlap between age-related t-map and anatomic brain regions. (C) Longitudinal changes (ΔSUVR) in [^18^F]MK6240 SUVR in putamen/pallidum did not present significant differences across groups, whereas cross-sectional SUVR was higher in CI individuals and lower in CUY group. ***P < 0.001. **P < 0.005. Δ = change calculated as $\frac{\left(\text{follow-up}\;\text{SUVR}\;-\;\text{baseline}\;\text{SUVR}\right)}{\text{time}\;(\text{y})};$ WM = white matter.

The average [^18^F]MK6240 SUVR images of CUY and CU Aβ− and tau-negative individuals less than 65 y old did not seem to display striking visual differences. However, a *t* test between the 2 groups revealed significantly higher [^18^F]MK6240 retention in the putamen, the pallidum, a parcel of cerebellar white matter, and a few other cortical regions (Fig. 3A). The same test was performed in the longitudinal sample as well (Supplemental Fig. 6). We assessed the percentage of overlap between the age-related signal and brain regions. The most important regional overlaps were with the putamen (75% of the region showing overlap) and the pallidum (72%), followed by the Braak stage II region (38%) (Fig. 3B). SUVRs in the putamen and pallidum differed significantly among diagnostic groups, with the CUY having a significantly lower [^18^F]MK6240 retention cross-sectionally (CUY vs. CU Aβ−, *P* < 0.001; CUY vs. CU Aβ+, *P* < 0.001; CUY vs. CI Aβ+, *P* < 0.001). Additionally, CI Aβ+ individuals had significantly higher values than CU individuals (Aβ− and Aβ+) cross-sectionally (CU Aβ− vs. CU Aβ+, *P* = 0.926; CU Aβ− vs. CI Aβ+, *P* < 0.001; CU Aβ+ vs. CI Aβ+, *P* < 0.001). Nevertheless, the longitudinal rate of change (ΔSUVR) did not present significant differences among the groups (CUY vs. CU Aβ−, *P* = 0.927; CUY vs. CU Aβ+, *P* = 0.845; CUY vs. CI Aβ+, *P* = 0.731; CU Aβ− vs. CU Aβ+, *P* = 0.880; CU Aβ− vs. CI Aβ+, *P* = 0.728; CU Aβ+ vs. CI Aβ+, *P* = 0.994] (Fig. 3C).

### Associations of Changes in Target, Age-Related, and Off-Target Retention

**FIGURE 4.**
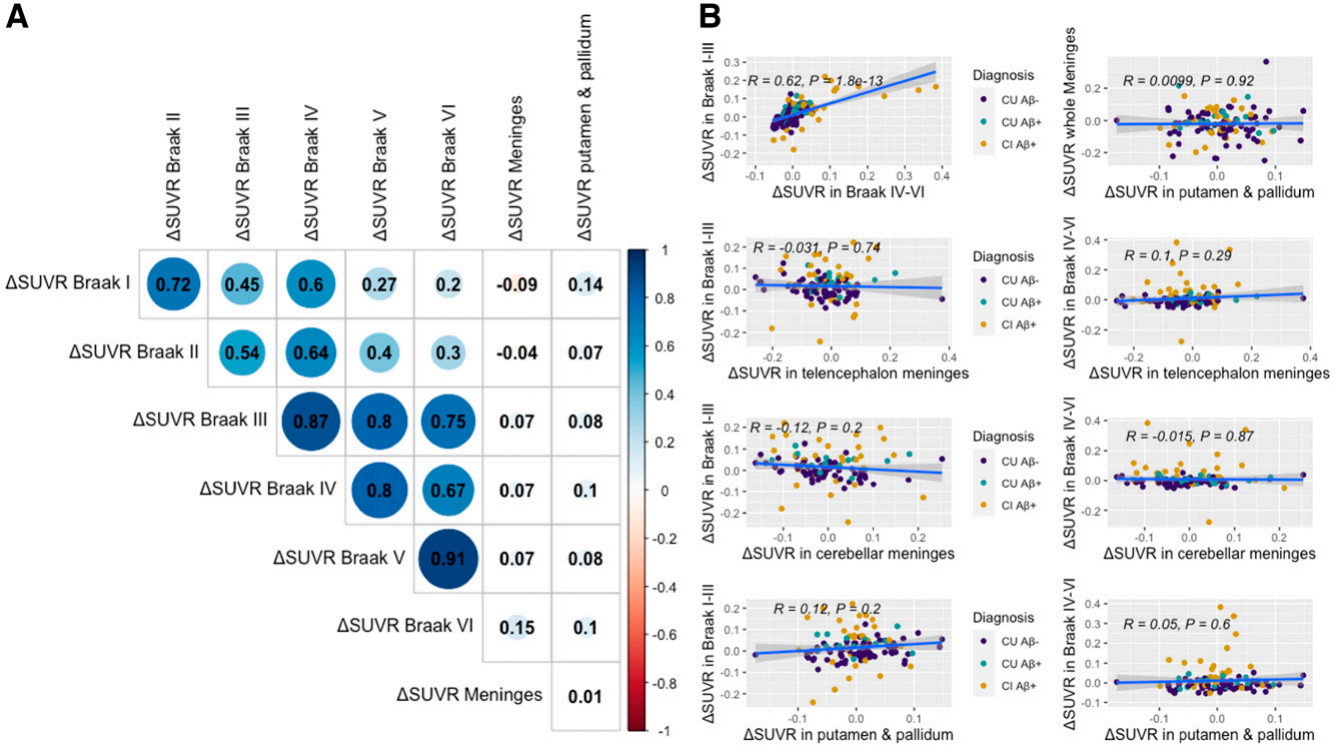
Correlations between longitudinal changes in SUVR in age-related, meningeal, and target regions. We assessed ΔSUVR in regions presenting target (Braak I–VI), off-target (both telencephalon and cerebellar meninges), and age-related (putamen/pallidum) tracer uptake. We observed strong ΔSUVR among target regions; however, those did not correlate with ΔSUVRs in off-target and age-related regions. Matrix (A) and plots (B) present estimates of Pearson correlations between regions. Δ = change calculated as $\frac{\left(\text{follow-up}\;\text{SUVR}\;-\;\text{baseline}\;\text{SUVR}\right)}{\text{time}\;(\text{y})}.$

Target regions were considered brain regions in which we expect to see [^18^F]MK6240 retention based on the pattern of tau distribution extensively reported in the postmortem literature (1*,*^27^). We found a strong correlation in ΔSUVR among target regions, with each Braak region being more strongly correlated with the adjacent stages. The weakest correlation was between the Braak I and Braak VI regions. When extracting the average SUVR in the Braak I–III and Braak IV–VI regions, we also observed a strong positive correlation between regions (*R* = 0.62, *P* < 0.001). We then used SUVRs in the telencephalon and cerebellar meninges, as well as in the putamen and pallidum, in the correlations. ΔSUVR in those regions did not correlate significantly with ΔSUVR in any one of the target regions (Braak I–III and telencephalon meninges: *R* = −0.03, *P* = 0.740; Braak IV–VI and telencephalon meninges: *R* = 0.10, *P* = 0.290; Braak I–III and cerebellar meninges: *R* = −0.12, *P* = 0.200; Braak IV–VI and cerebellar meninges: *R* = −0.02, *P* = 0.870; Braak I–III and putamen and pallidum: *R* = 0.12, *P* = 0.200; Braak IV–VI and putamen and pallidum: *R* = 0.05, *P* = 0.600). Nor did the meningeal and putamen/pallidum ΔSUVR correlate with each other (*R* = 0.01, *P* = 0.920) (Fig. 4). To further assess the impact of meninges on tracer quantification, we categorized individuals as having high or low meningeal retention (based on SUV median for meninges). We did not find difference in diagnostic groups or longitudinal tracer accumulation between individuals with high and low meningeal retention (Supplemental Table 6). Finally, we assessed the stability of [^18^F]MK6240 SUVR in target regions over time when using different reference regions (i.e., inferior CG, crus I, full CG, and superior CG). We extracted Braak IV–VI SUVRs in CUY and CU Aβ− individuals, for whom we do not expect a significant increase in SUVRs. We observed no difference between baseline and follow-up values when using either reference region (Supplemental Fig. 7; Supplemental Table 7).

## DISCUSSION

This study suggested that the most widely used cerebellar reference regions (inferior CG, superior CG, crus I, and full CG) present stability, with no changes over time, and therefore may be suitable for use in longitudinal studies, although differences were observed in the superior CG and crus I cross-sectionally. We found evidence for the existence of an age-related retention in the putamen/pallidum, similar—albeit of much lower magnitude—to the reported off-target retention observed using the first-generation tau PET tracers (12*,*^28^). Finally, we demonstrated that there was no association between [^18^F]MK6240 ΔSUVR in target regions and in age-related or meningeal off-target signals over the time frame of our study.

Previous cross-sectional studies have already shown that indices of tau load made using [^18^F]MK6240 are amenable to simplified tissue ratio methods using data acquired 90–110 min after injection (4*,*^7^). However, questions remain regarding the suitability of the reference region for longitudinal tracer quantification because of the bias often inherent in SUVR data (10). This is of paramount importance because [^18^F]MK6240 has been used in clinical trial settings to capture longitudinal changes in tau tangle pathology (29). Extensive research has focused on the cerebellum as the appropriate reference region for tau radiotracers (30*,*^31^), as the CG is not expected to harbor significant NFT pathology (1). Although the gold standard method for assessment of the optimal reference region relies on dynamic quantifications with arterial input function, one crucial characteristic of a reference region for longitudinal assessments is not having large variability over time, across diagnostic groups, or in other pathologic features (i.e., Aβ status in the case of AD research) (32). In this study, we estimated the [^18^F]MK6240 SUVs in distinct regions of the CG at baseline, as well as its change over time, based on diagnosis and on Aβ status. The results indicate that there were small cross-sectional differences between diagnostic groups in the superior CG and crus I but not in the inferior CG and full CG. These differences might be due to spillover effect from the target regions, as individuals with AD dementia inherently have a higher uptake of the tracer. Additionally, we did not observe differences in cerebellar SUVs based on Aβ status. When examining the differences between baseline and follow-up assessments, we did not observe any significant difference in [^18^F]MK6240 SUV for any cerebellar region. Even though all variability was relatively minor, we observed the lowest numeric variability in the SUV levels of the inferior CG cross-sectionally and in the crus I longitudinally. Altogether, our results suggest that tracer retention in the tested reference regions was relatively stable over time and across diagnostic groups, suggesting that all these reference regions could potentially be used for longitudinal [^18^F]MK6240 quantification. Given the cross-sectional differences in tracer uptake among diagnostic groups in the superior CG and crus I but not in the inferior CG, this latter region was deemed more appropriate for the cross-sectional and longitudinal [^18^F]MK6240 quantification and thus was used for the remaining analyses. Future studies using the gold standard arterial input function should address other important characteristics of an optimal reference region.

The *t* test comparing young individuals under age 35 y and participants between 40 and 65 y old allowed us to assess age-related retention of [^18^F]MK6240. The regions presenting the higher age-related [^18^F]MK6240 retention were the putamen and pallidum. Those are often considered off-target regions using other radiotracers for tau (31–33), but the retention seems to be of lower magnitude with [^18^F]MK6240 (34). As we included participants younger than 65 y who were CU Aβ− and tau-negative, we do not expect on-target [^18^F]MK6240 retention in subcortical structures based on the postmortem literature (35). Indeed, subcortical regions have been shown to harbor NFT accumulation only at late Braak pathologic stages (27*,*^36^), which might explain why we observed a significant cross-sectional difference in putamen and pallidum SUVR between CI Aβ+ individuals and CU individuals (either Aβ− or Aβ+). Similar to first-generation tau PET tracers (5), the retention observed with [^18^F]MK6240 in the putamen and pallidum may be due to neuromelanin deposition. Although the tracer retention observed in the Braak II region can represent an age-related signal, we cannot entirely exclude that there is some true concentration of NFTs in this region, as modest tau accumulation in CU Aβ− individuals has already been reported in the hippocampus (2*,*^35^). Another possibility is that the marked off-target retention of first-generation tracers in the choroid plexus (12), which contaminates the Braak II region for these tracers, may be a minor age-related problem with [^18^F]MK6240 as well. However, it is important to note that some individuals may have primary age-related tauopathy (37) and may be harboring NFT accumulation in early Braak stages, with little to no Aβ deposition. Finally, we assessed meningeal retention in both the telencephalon and the cerebellar regions, which have already been characterized as off-target by previous postmortem studies (5). Importantly, we observed no significant difference in the magnitude of meningeal uptake across diagnosis and Aβ status and no change over time. Nevertheless, we observed sex differences in meningeal retention cross-sectionally but not longitudinally, as previously reported for other tau PET tracers in the meninges and skull (13). Taken together, these results suggest that besides the meningeal retention, [^18^F]MK6240 presents a newly described age-related retention in subcortical brain regions, the cause of which needs to be elucidated by future in vitro studies across the aging spectrum.

We observed no association between annualized [^18^F]MK6240 SUVR changes in target, age-related regions, and meninges. Braak regions were used to represent target areas for tau tangles, as extensively reported in postmortem studies (1*,*^27^). ΔSUVRs in target brain regions correlated strongly with each other, suggesting that changes in tracer retention in these brain regions are influenced by the same brain process, likely NFT accumulation (22). On the other hand, we did not observe a significant correlation between ΔSUVR in meningeal off-target uptake and changes in Braak target regions. Additionally, age-related ΔSUVR in the putamen and pallidum did not correlate with that in target regions. Nor did off-target, meningeal, and age-related subcortical ΔSUVR correlate with each other. This finding suggests that different processes set the pace of progression in target, off-target, and age-related regions and that spillover from off-target regions would not heavily influence rates of progression in [^18^F]MK6240 target regions.

This study had limitations. The lack of arterial sampling at baseline and follow-up limits assessments of reference region and accurate tracer retention. We observed a small (close to zero), non–statistically significant decrease in SUVs in cerebellar regions over time. Additionally, all [^18^F]MK6240 analyses were conducted using images acquired from 90 to 110 min after injection. Even though this simplified quantitative approach has been validated (4), dynamically acquired PET data with arterial input function would be more appropriate to test the hypotheses of our study. Although there is a possibility that the effect of meninges in reference regions may lead to changes in baseline and follow-up values and, consequently, in rates of change, the fact that meningeal uptake did not change significantly over time or differ between groups defined using cognition or Aβ status suggests that it does not play a major role in the longitudinal results for this tracer. Moreover, we used spatial smoothing of 8 mm; smaller smoothing would likely cause the meninges to have less impact on the adjacent brain regions. An additional limitation is the lack of partial-volume correction in our study. Postmortem data would validate our findings, as such data would allow us to ensure the absence of NFTs in the cerebellar regions, as well as in the age-related retention regions. Without postmortem confirmation, we cannot exclude that age-related retention in CU adults is caused by tau tangle pathology. Moreover, our sample was restricted to a follow-up of 1.5–3.5 y; using other follow-up durations may give different results. We evaluated only a small subset of reference regions that are frequently reported in the literature; other regions may present better results for [^18^F]MK6240 longitudinal quantification. An additional limitation is that we did not provide any evidence about the mechanism through which age-related retention occurs. Arterial and postmortem data are needed to understand our findings.

## CONCLUSION

The inferior CG is a suitable reference region for cross-sectional and longitudinal quantification of [^18^F]MK6240. [^18^F]MK6240 exhibits off-target retention in the meninges and an age-related signal in the putamen and pallidum, also likely representing off-target retention, and in the Braak II region, for which the source needs to be elucidated. The lack of an association between changes in SUVR within age-related, off-target, and target regions suggests that longitudinal changes in [^18^F]MK6240 are not heavily driven by changes in age-related or off-target signals. However, future postmortem studies are needed to clarify these findings.

## DISCLOSURE

This work was supported by the Weston Brain Institute, Fonds de Recherche Santé Québec, Healthy Brain for Healthy Lives, and the McGill University Faculty of Medicine. Tharick Pascoal is supported by the Alzheimer Association (AACSF-20-648075) and the National Institutes of Health (R01AG073267 and R01AG075336). Serge Gauthier has served as a scientific advisor to Cerveau Therapeutics. No other potential conflicts of interest relevant to this article exist.

## Notes

KEY POINTS **QUESTION:** What is the effect of reference region and nontarget tracer retention on longitudinal quantification of [^18^F]MK6240 in target regions? **PERTINENT FINDINGS:** The inferior CG and full CG are appropriate reference regions for cross-sectional and longitudinal quantification of [^18^F]MK6240. This tracer is already known to present off-target binding in the meninges, but we discovered a so-called age-related binding in the putamen and pallidum. However, the longitudinal changes in nontarget tracer retention did not correlate with longitudinal changes in on-target regions. **IMPLICATIONS FOR PATIENT CARE:** [^18^F]MK6240 can be used as a surrogate marker in clinical trials, as long as the appropriate reference region is used and off-target and age-related retention are considered.
